# Supplementary material for: An open-source framework for large-scale, flexible evaluation of biomedical text mining systems
Source: J Biomed Discov Collab. 2008 Jan 29;3:1. doi: 10.1186/1747-5333-3-1 (PMC2276192; doi:10.1186/1747-5333-3-1)
Supplement: Additional file 1 — Evaluation platform source code. The additional file contains the software evaluation framework discussed in this paper. The most current release can be downloaded from SourceForge.net [1]. See the accompanying README file for instructions on installation and use. Also included in the distribution is a collection of UIMA wrappers for some commonly used BioNLP tools and annotated corpora. [file 1747-5333-3-1-S1.zip › bionlp_uima_repository-1.0.1/docs/api/src-html/edu/uchsc/ccp/uima/annotation/CCPDocumentInformation.html]

```
001    
002    
003    /* First created by JCasGen Tue Aug 28 16:44:10 MDT 2007 */
004    package edu.uchsc.ccp.uima.annotation;
005    
006    import org.apache.uima.jcas.JCas;
007    import org.apache.uima.jcas.JCasRegistry;
008    import org.apache.uima.jcas.cas.StringArray;
009    import org.apache.uima.jcas.cas.TOP_Type;
010    
011    
012    /** The CCPDocumentInformation annotation includes document metadata such as the document ID, document collection ID, secondary document IDs, document size, etc.
013     * Updated by JCasGen Tue Aug 28 16:44:10 MDT 2007
014     * XML source: /home/williamb/eclipse/RELEASED_SOURCE_CODE/descriptors/typeSystem/CCPTypeSystem.xml
015     * @generated */
016    public class CCPDocumentInformation extends CCPDocumentAnnotation {
017      /** @generated
018       * @ordered 
019       */
020      public final static int typeIndexID = JCasRegistry.register(CCPDocumentInformation.class);
021      /** @generated
022       * @ordered 
023       */
024      public final static int type = typeIndexID;
025      /** @generated  */
026      public              int getTypeIndexID() {return typeIndexID;}
027     
028      /** Never called.  Disable default constructor
029       * @generated */
030      protected CCPDocumentInformation() {}
031        
032      /** Internal - constructor used by generator 
033       * @generated */
034      public CCPDocumentInformation(int addr, TOP_Type type) {
035        super(addr, type);
036        readObject();
037      }
038      
039      /** @generated */
040      public CCPDocumentInformation(JCas jcas) {
041        super(jcas);
042        readObject();   
043      } 
044    
045      /** @generated */  
046      public CCPDocumentInformation(JCas jcas, int begin, int end) {
047        super(jcas);
048        setBegin(begin);
049        setEnd(end);
050        readObject();
051      }   
052    
053      /** <!-- begin-user-doc -->
054        * Write your own initialization here
055        * <!-- end-user-doc -->
056      @generated modifiable */
057      private void readObject() {}
058         
059     
060        
061      //*--------------*
062      //* Feature: documentID
063    
064      /** getter for documentID - gets The document ID is a String representing a unique identifier for a particular document within a particular document collection.
065       * @generated */
066      public String getDocumentID() {
067        if (CCPDocumentInformation_Type.featOkTst && ((CCPDocumentInformation_Type)jcasType).casFeat_documentID == null)
068          jcasType.jcas.throwFeatMissing("documentID", "edu.uchsc.ccp.uima.annotation.CCPDocumentInformation");
069        return jcasType.ll_cas.ll_getStringValue(addr, ((CCPDocumentInformation_Type)jcasType).casFeatCode_documentID);}
070        
071      /** setter for documentID - sets The document ID is a String representing a unique identifier for a particular document within a particular document collection. 
072       * @generated */
073      public void setDocumentID(String v) {
074        if (CCPDocumentInformation_Type.featOkTst && ((CCPDocumentInformation_Type)jcasType).casFeat_documentID == null)
075          jcasType.jcas.throwFeatMissing("documentID", "edu.uchsc.ccp.uima.annotation.CCPDocumentInformation");
076        jcasType.ll_cas.ll_setStringValue(addr, ((CCPDocumentInformation_Type)jcasType).casFeatCode_documentID, v);}    
077       
078        
079      //*--------------*
080      //* Feature: documentCollectionID
081    
082      /** getter for documentCollectionID - gets The document collection ID is an Integer that uniquely identifies a particular document collection.
083       * @generated */
084      public int getDocumentCollectionID() {
085        if (CCPDocumentInformation_Type.featOkTst && ((CCPDocumentInformation_Type)jcasType).casFeat_documentCollectionID == null)
086          jcasType.jcas.throwFeatMissing("documentCollectionID", "edu.uchsc.ccp.uima.annotation.CCPDocumentInformation");
087        return jcasType.ll_cas.ll_getIntValue(addr, ((CCPDocumentInformation_Type)jcasType).casFeatCode_documentCollectionID);}
088        
089      /** setter for documentCollectionID - sets The document collection ID is an Integer that uniquely identifies a particular document collection. 
090       * @generated */
091      public void setDocumentCollectionID(int v) {
092        if (CCPDocumentInformation_Type.featOkTst && ((CCPDocumentInformation_Type)jcasType).casFeat_documentCollectionID == null)
093          jcasType.jcas.throwFeatMissing("documentCollectionID", "edu.uchsc.ccp.uima.annotation.CCPDocumentInformation");
094        jcasType.ll_cas.ll_setIntValue(addr, ((CCPDocumentInformation_Type)jcasType).casFeatCode_documentCollectionID, v);}    
095       
096        
097      //*--------------*
098      //* Feature: documentSize
099    
100      /** getter for documentSize - gets The size of a document is logged as the number of characters it contains.
101       * @generated */
102      public int getDocumentSize() {
103        if (CCPDocumentInformation_Type.featOkTst && ((CCPDocumentInformation_Type)jcasType).casFeat_documentSize == null)
104          jcasType.jcas.throwFeatMissing("documentSize", "edu.uchsc.ccp.uima.annotation.CCPDocumentInformation");
105        return jcasType.ll_cas.ll_getIntValue(addr, ((CCPDocumentInformation_Type)jcasType).casFeatCode_documentSize);}
106        
107      /** setter for documentSize - sets The size of a document is logged as the number of characters it contains. 
108       * @generated */
109      public void setDocumentSize(int v) {
110        if (CCPDocumentInformation_Type.featOkTst && ((CCPDocumentInformation_Type)jcasType).casFeat_documentSize == null)
111          jcasType.jcas.throwFeatMissing("documentSize", "edu.uchsc.ccp.uima.annotation.CCPDocumentInformation");
112        jcasType.ll_cas.ll_setIntValue(addr, ((CCPDocumentInformation_Type)jcasType).casFeatCode_documentSize, v);}    
113       
114        
115      //*--------------*
116      //* Feature: secondaryDocumentIDs
117    
118      /** getter for secondaryDocumentIDs - gets This StringArray is used for secondary document ID storage. For example, in the biomedical domain, a particular document might be associated with a PubMed ID, however it might also have a deprecated Medline ID, or perhaps a PubMed Central ID, either of which could be stored in this StringArray. It is recommended that the type of ID along with the ID itself be stored, e.g. "MedlineID:12345".
119       * @generated */
120      public StringArray getSecondaryDocumentIDs() {
121        if (CCPDocumentInformation_Type.featOkTst && ((CCPDocumentInformation_Type)jcasType).casFeat_secondaryDocumentIDs == null)
122          jcasType.jcas.throwFeatMissing("secondaryDocumentIDs", "edu.uchsc.ccp.uima.annotation.CCPDocumentInformation");
123        return (StringArray)(jcasType.ll_cas.ll_getFSForRef(jcasType.ll_cas.ll_getRefValue(addr, ((CCPDocumentInformation_Type)jcasType).casFeatCode_secondaryDocumentIDs)));}
124        
125      /** setter for secondaryDocumentIDs - sets This StringArray is used for secondary document ID storage. For example, in the biomedical domain, a particular document might be associated with a PubMed ID, however it might also have a deprecated Medline ID, or perhaps a PubMed Central ID, either of which could be stored in this StringArray. It is recommended that the type of ID along with the ID itself be stored, e.g. "MedlineID:12345". 
126       * @generated */
127      public void setSecondaryDocumentIDs(StringArray v) {
128        if (CCPDocumentInformation_Type.featOkTst && ((CCPDocumentInformation_Type)jcasType).casFeat_secondaryDocumentIDs == null)
129          jcasType.jcas.throwFeatMissing("secondaryDocumentIDs", "edu.uchsc.ccp.uima.annotation.CCPDocumentInformation");
130        jcasType.ll_cas.ll_setRefValue(addr, ((CCPDocumentInformation_Type)jcasType).casFeatCode_secondaryDocumentIDs, jcasType.ll_cas.ll_getFSRef(v));}    
131        
132      /** indexed getter for secondaryDocumentIDs - gets an indexed value - This StringArray is used for secondary document ID storage. For example, in the biomedical domain, a particular document might be associated with a PubMed ID, however it might also have a deprecated Medline ID, or perhaps a PubMed Central ID, either of which could be stored in this StringArray. It is recommended that the type of ID along with the ID itself be stored, e.g. "MedlineID:12345".
133       * @generated */
134      public String getSecondaryDocumentIDs(int i) {
135        if (CCPDocumentInformation_Type.featOkTst && ((CCPDocumentInformation_Type)jcasType).casFeat_secondaryDocumentIDs == null)
136          jcasType.jcas.throwFeatMissing("secondaryDocumentIDs", "edu.uchsc.ccp.uima.annotation.CCPDocumentInformation");
137        jcasType.jcas.checkArrayBounds(jcasType.ll_cas.ll_getRefValue(addr, ((CCPDocumentInformation_Type)jcasType).casFeatCode_secondaryDocumentIDs), i);
138        return jcasType.ll_cas.ll_getStringArrayValue(jcasType.ll_cas.ll_getRefValue(addr, ((CCPDocumentInformation_Type)jcasType).casFeatCode_secondaryDocumentIDs), i);}
139    
140      /** indexed setter for secondaryDocumentIDs - sets an indexed value - This StringArray is used for secondary document ID storage. For example, in the biomedical domain, a particular document might be associated with a PubMed ID, however it might also have a deprecated Medline ID, or perhaps a PubMed Central ID, either of which could be stored in this StringArray. It is recommended that the type of ID along with the ID itself be stored, e.g. "MedlineID:12345".
141       * @generated */
142      public void setSecondaryDocumentIDs(int i, String v) { 
143        if (CCPDocumentInformation_Type.featOkTst && ((CCPDocumentInformation_Type)jcasType).casFeat_secondaryDocumentIDs == null)
144          jcasType.jcas.throwFeatMissing("secondaryDocumentIDs", "edu.uchsc.ccp.uima.annotation.CCPDocumentInformation");
145        jcasType.jcas.checkArrayBounds(jcasType.ll_cas.ll_getRefValue(addr, ((CCPDocumentInformation_Type)jcasType).casFeatCode_secondaryDocumentIDs), i);
146        jcasType.ll_cas.ll_setStringArrayValue(jcasType.ll_cas.ll_getRefValue(addr, ((CCPDocumentInformation_Type)jcasType).casFeatCode_secondaryDocumentIDs), i, v);}
147       
148        
149      //*--------------*
150      //* Feature: classificationType
151    
152      /** getter for classificationType - gets This String provides a means for classifying a particular document.
153       * @generated */
154      public String getClassificationType() {
155        if (CCPDocumentInformation_Type.featOkTst && ((CCPDocumentInformation_Type)jcasType).casFeat_classificationType == null)
156          jcasType.jcas.throwFeatMissing("classificationType", "edu.uchsc.ccp.uima.annotation.CCPDocumentInformation");
157        return jcasType.ll_cas.ll_getStringValue(addr, ((CCPDocumentInformation_Type)jcasType).casFeatCode_classificationType);}
158        
159      /** setter for classificationType - sets This String provides a means for classifying a particular document. 
160       * @generated */
161      public void setClassificationType(String v) {
162        if (CCPDocumentInformation_Type.featOkTst && ((CCPDocumentInformation_Type)jcasType).casFeat_classificationType == null)
163          jcasType.jcas.throwFeatMissing("classificationType", "edu.uchsc.ccp.uima.annotation.CCPDocumentInformation");
164        jcasType.ll_cas.ll_setStringValue(addr, ((CCPDocumentInformation_Type)jcasType).casFeatCode_classificationType, v);}    
165      }
166    
167
```
